# Assessment of the Broader Economic Consequences of HPV Prevention from a Government-Perspective: A Fiscal Analytic Approach

**DOI:** 10.1371/journal.pone.0160707

**Published:** 2016-08-04

**Authors:** Didik Setiawan, Nikolaos Kotsopoulos, Jan C. Wilschut, Maarten J. Postma, Mark P. Connolly

**Affiliations:** 1 Unit of PharmacoEpidemiology & PharmacoEconomics (PE2), Department of Pharmacy, University of Groningen, Groningen, The Netherlands; 2 Faculty of Pharmacy, University of Muhammadiyah Purwokerto, Purwokerto, Indonesia; 3 Global Market Access Solutions (GMAS), St-Prex, Switzerland; 4 Department of Medical Microbiology, University Medical Center Groningen, University of Groningen, Groningen, The Netherlands; 5 Institute of Science in Healthy Aging & healthcaRE (SHARE), University Medical Center Groningen (UMCG), Groningen, The Netherlands; Bharathidasan University, INDIA

## Abstract

**Background:**

Cervical cancer poses a substantial burden in terms of morbidity, mortality, and economic losses, especially in low/middle-income countries. HPV vaccination and/or cervical cancer screening among females may reduce the burden of HPV-related diseases, including cervical cancer. However, limited funds may impede the implementation of population-based programmes. Governmental investments in the prevention of infectious disease may have broader economic and fiscal benefits, which are not accounted in conventional economic analyses. This study estimates the broader economic and fiscal impacts of implementing HPV vaccination and/or cervical cancer screening in Indonesia from the perspective of the government.

**Methods:**

A government-perspective quantitative analytic framework was applied to assess the Net Present Value (NPV) of investment on cervical cancer prevention strategies including HPV vaccination, cervical screening and its combination in Indonesia. All monetary values were presented in International Dollars (I$).

**Results:**

Based on a cohort of 10,000,000 Indonesian 12-year-old females, it was estimated that HPV vaccination and/or cervical cancer screening result in a positive NPV for the Indonesian government. The combination of cervical screening and HPV vaccination generated a substantial reduction of cervical cancer incidence and HPV-related mortality of 87,862 and 19,359, respectively. It was estimated that HPV vaccination in combination with cervical screening is the most favorable option for cervical cancer prevention (NPV I$2.031.786.000), followed by HPV vaccination alone (NPV I$1.860.783.000) and cervical screening alone (NPV I$375.244.000).

**Conclusion:**

In addition to clinical benefits, investing in HPV vaccination and cervical screening may yield considerable fiscal benefits for the Indonesian governments due to lifelong benefits resulting from reduction of cervical cancer-related morbidity and mortality.

## Introduction

High-risk human papillomaviruses (hrHPV), in particular HPV16 and HPV18, are responsible for cervical cancer and premalignant cervical disease among woman [1–5]. Although cervical cancer is considered as a preventable disease when detected and managed in the early stages, it still poses a serious health and economic burden, especially in developing countries [6–8]. The WHO recommends primary prevention through vaccination against HPV and secondary prevention through cervical screening for pre-cancerous lesions [9]. Numerous studies have shown that cervical screening is beneficial in terms of lowering the incidence of pre-cancerous lesions and cervical cancer [8,10,11]. Moreover, the two approved prophylactic vaccines against HPV have also demonstrated considerable benefits in terms of reduction of HPV infection and Cervical Intraepithelial Neoplasia (CIN) [9,12–15].

Several health-economic analyses have shown that HPV vaccination combined with cervical cancer screening results in an efficient use of healthcare resources [16–19]. Such analyses typically assess the cost-effectiveness of adding HPV vaccination to screening. Hence, most economic analyses focus on evaluating the technical efficiency of the healthcare budget [20]. Implementation of vaccination and cervical cancer screening programmes is dependent on affordability and local infrastructure especially in low/middle-income countries, which have limited infrastructure, human resources and funds. In low/middle-income countries, implementation of population-based HPV vaccination and screening programmes, such as VIA screening which is suitable for low/middle-income countries, may require funds additional to the healthcare budget. Hence, economic analyses should be capable of providing decision-makers with measures for the broader economic impact and cross-sectorial consequences of resource allocation decisions.

HPV infection and cervical cancer disease pose a substantial morbidity and mortality burden. In 2012, there were over 20,000 new cases of cervical cancer and 9,000 cervical-cancer related deaths [7]. The implementation of a population-based HPV vaccination and/or VIA screening programme requires a substantial investment, but it potentially reduces the burden of cervical cancer. An additional benefits including more women who are able to work, more women gain income [21–23], and on the government perspective, there will be more workers who pay the taxes and health/social security premium. Notably, health-economic and broader economic benefits including fiscal benefits for the Indonesian government could be achieved. In this study, we assess the broader economic consequences of introducing HPV vaccination in Indonesia with emphasis on the potential positive and negative fiscal effects of the latest policy of the Indonesian National Social Security System (*SJSN*) for HPV prevention.

## Methods and Data

The natural history of cervical cancer for Indonesian women was modeled based on a previously published population-based Markov model [24]. The cervical cancer prevention strategies, including HPV vaccination (3 doses of vaccination and 76.6% coverage) and/ or VIA screening (every three years for 30–60 years old and 21.2% yearly coverage), were also adopted from the same study. An additional strategy, HPV vaccination alone, was also added in the analysis. This population-based Markov model was chosen given the scarce availability of data, hampering the development of a more complex modeling approach. Several validation steps were taken, including face validity checks for both the conceptual model and the input parameters, extreme value testing and testing of traces.

The fiscal analysis described here simulates the natural history and costs of cervical cancer patients in Indonesia. Susceptible, cervical cancer and death states were modeled and a simulation was run for a cohort of 10,000,000 Indonesian 12-year-old girls over their lifetime [25,26]. Cervical cancer prevention strategies, that were assessed, included (i) cervical screening, (ii) HPV vaccination and (iii) HPV vaccination plus cervical cancer screening. The reduction of HPV-related morbidity and mortality resulting from each of the assessed strategies was quantified and translated into changes in age-specific gross tax revenue and government transfer costs over the lifetime of the cohort. Specifically, based on previously published methods for assessing of the broader economic consequences of health-care interventions [27–29], morbidity and mortality reductions were translated into discounted, lifetime fiscal benefits for the Indonesian government. Hence, prevention of HPV-related mortality and morbidity benefits were converted into discounted lifetime (i) additional gross, direct and indirect tax revenues, (ii) health-care cost savings and (iii) social insurance cost-savings. Fiscal expenses, i.e. the cost of vaccination and the additional governmental transfers (i.e. pensions) resulting from the additional survival produced by HPV prevention in Indonesia, were discounted and deducted from the fiscal benefits of HPV prevention to produce the Net Present Value (NPV) of each HPV prevention strategy.

In order to estimate the lifetime earnings and, thus, the lifetime tax revenues to be gained from the average Indonesian female, earnings distribution was categorized into three levels (20% high-income earners, 40% middle-income earners, and 40% low-income earners) and the average of each earnings level, based on the Indonesian Gross Domestic Product (GDP) using Gini index of 0.41 [30,31], was estimated. Furthermore, annual earnings were adjusted for future productivity based on the labor productivity index from The Organization for Economic Cooperation and Development (OECD) [32]. Earnings were also adjusted to reflect the proportion of female workforce participation [25,33]. Moreover, the average age of entering the workforce and the average retirement age of Indonesian civil servants were considered in the model [34,35]. Direct tax was estimated based on the Indonesian earnings tax law [36,37] and applied for each level of income. Thus, the model assumed that tax revenue stems from middle- and high-income earnings only, since the average earnings of the low-income population lies below the taxable earnings threshold. Indirect tax was estimated based on the Value Added Tax (VAT) policy [38,39] and household consumption expenditure per capita [40]. Furthermore, we assumed that VAT adherence and collectability rate was 50% [41] (Table 1).

**Table 1:** Model input Parameters.

Type	Description	value	Comments	Reference
**National economic parameters**	Life expectancy	70.10		[42]
	Gross Domestic Products (I$)	7,463	per capita	[31]
	Average Earnings(I$)			
	Low	3,183	as percentage of national GDP	[30,31]
	Middle	6,375	as percentage of national GDP	[30,31]
	High	18,198	as percentage of national GDP	[30,31]
	Retirement age	60		[35]
	Annual inflation (%)	6.42	inflation rate	[43]
	Civil servant (%)	2.00	as percentage of female population	[33]
	Minimum age as a civil servant	18		[34]
	Labor Productivity (%)	3.60	Yearly	[32]
	Discount rate (%)	3.00		
**Revenue**	Direct tax (%)		based on Tax earnings bands	
	< I$6,390	0.00	as percentage of income	[36,37]
	I$6,390—I$13,150	5.00	as percentage of income	[36,37]
	I$13,150—I$65,748	15.00	as percentage of income	[36,37]
	Indirect tax			
	VAT (%)	10.00	value added tax	[38,39]
	Earnings consumed (I$)	1,960	household consumption expenditure per capita	[40]
	Tax adherence (%)	50.00	Assumption	-
	Social security			
	Pension fund benefit (%)			
	Employee	2.00	as percentage of income	[44,45]
	Employer	3.70	as percentage of income	[44,45]
	Net revenue	47.10	after deducted by claim	[46]
	Work-related accident benefit (%)			
	Employer	0.90	as percentage of income	[44,45]
	Net revenue	71.00	after deducted by claim	[46]
	Death benefit (%)			
	Employer	0.30	as percentage of income	[44,45]
	Net revenue	60.00	after deducted by claim	[46]
	Membership of social security (%)	75.00		[47]
	Health security (%)		based on the participant's type	
	Employee (civil servants)	2.00	as percentage of income	[44,45]
	Employee (non-civil servants)	2.00	as percentage of income	[44,45]
	Employer (non-civil servants)	3.00	as percentage of income	[44,45]
	Membership of health security (%)	75.00		[47]
**Expenditure**	Social security for civil servant (%)	6.00		
	Pension fund benefit			
	Employer	3.70	as percentage of income	[44,45]
	Work related accident benefit			
	Employer	0.90	as percentage of income	[44,45]
	Death benefit			
	Employer	0.30	as percentage of income	[44,45]
	Death benefit paid (I$)	5	per case	[44,45]
	Health security		based on the participant's type	
	Poor people (I$)	5	per person	[44,45]
	Employer (%)	3.00	as percentage of income	[44,45]
	Pension Benefit (%)	60.00		[48]

Governmental transfers to citizens were estimated based on the national social and health security system. The benefits provided by the social security system are pension funds, and work-related accident and death-benefits [44,45]. Old-age pensions were estimated based as 60% of the earnings in the last employment year [48]. Direct costs related to pre-cancerous lesions, cervical cancer and investment costs for prevention programs were included in the model based on the study by Setiawan, *et al*. [24]. Costs were inflated to current prices using an inflation rate of 6.42% which reflects the average inflation in 2014 [43]. Direct costs attributed to genital warts have not been included in this analysis as they are likely to have limited fiscal consequences in the context of treatment in Indonesia.

The discounted cash flow method was used to obtain the present value of cash inflows and outflows. The results were presented in terms of the Net Present Value (NPV) of the investment decision for each of the cervical cancer prevention strategies under study as follows:

$$NPV=\sum_{t=0}^{T}(\frac{R_{t}-E_{t}}{{\left(1+r\right)}^{t}})-K_{0}$$

NPVs were calculated by subtracting incremental expenses (E_t_), including vaccine cost at t = 0 (K_0_) from incremental revenues (R_t_) for each of strategy. Increments refer to the difference between the revenue and expenses with and without the HPV prevention strategy under consideration. All values were discounted (r) by 3% according to WHO recommendation and also consistent with previous cost-effectiveness studies on cervical cancer prevention programmes in Indonesia.

In order to examine the sensitivity of the model to its parameters, univariate sensitivity analyses were conducted for the NPV of each HPV prevention strategy. The ranges around input parameters including inflation rate, discount rate, Gross domestic products (GDP), tax compliance, membership of health and social security, percentage of earnings consumed and percentage of work-related accident premium. Inflation and discount rates were varied by the lowest and highest value of Indonesian inflation rate in 2014 [43] and acceptable discount rates from various countries [49], respectively. The lower and upper limits for GDP were defined by the lowest and highest GDP value of the previous 5 years [50], while the ranges for the proportion of earnings consumed were based on previous 3 years’ data [51]. Tax compliance, health and social security membership were varied based on assumptions since the distributions for this parameters were not available.

## Results

Based on a single cohort of 10,000,000 girls in Indonesia, our model showed that in the absence of any cervical cancer prevention strategy, 147,632 cases of cervical cancer will ultimately occur within the cohort. The implementation of cervical cancer prevention strategies, including screening alone, HPV vaccination alone, or cervical screening in combination with HPV vaccination were projected to prevent 15,641, 80,750 or 87,862 cases of cervical cancer, respectively. Furthermore, the combination of cervical screening and HPV vaccination generated the highest reduction of deaths caused by cervical cancer (19,359), followed by HPV vaccination alone (17,541) and screening alone (4,005) (Table 2).

**Table 2:** The impact of cervical cancer prevention strategies on incidence and mortality cases over a 58-years period.

Clinical Parameters	No Intervention	Screening	Cervical Screening & HPV Vaccination	HPV Vaccination
**Cumulative incidence**	147,632	131,991	59,770	66,882
**Incremental incidence**	-	15,641	87,862	80,750
**Cumulative mortality**	32,046	28,041	12,687	14,505
**Incremental mortality**	-	4,005	19,359	17,541

The lifetime NPV for the study cohort of Indonesian 12-year-old females is illustrated in Table 3 for the three prevention strategies. All three strategies resulted in a positive NPV. HPV vaccination in combination with cervical screening generates the highest NPV for the government (I$2,031,786,000), followed by HPV vaccination only (I$1,860,783,000) and cervical screening alone (I$375,244,000). Indirect tax produces the highest revenue for the government compared to direct tax, social- or health-security contributions. With respect to HPV-related governments’ expenses, the highest saving is generated by treatment cost savings. HPV vaccination in combination with cervical cancer screening results in the highest healthcare cost-savings. Similarly, the combined prevention strategy resulted in the highest savings in terms of social security costs. All prevention strategies resulted in increased governments’ expenses in terms of health security and pension costs.

**Table 3:** The impact of cervical cancer prevention strategies on future governments’ revenue and expenses (x I$1,000; year 2014).

Strategy	Revenue				Gross Tax Revenue per strategy	Transfers				Total Transfers	Net Present Value (NPV
	Direct Tax	Indirect Tax	Social Security	Health Security		Social Security	Health Security	pension	Treatment Cost		
**No Intervention**	273,405,393	125,213,166	67,387,009	79,908,077	**545,913,646**	7,468	290,926	363,881,820	4,040,308	**368,220,522**	**177,693,124**
**Cervical Screening**	273,405,656	125,213,470	67,387,071	79,908,151	**545,914,348**	7,440	290,926	363,885,561	3,662,052	**367,845,980**	**178,068,368**
**Cervical screening and HPV vaccination**	273,406,098	125,214,083	67,387,175	79,908,274	**545,915,630**	7,344	290,926	363,893,627	1,998,824	**366,190,721**	**179,724,910**
**HPV Vaccination**	273,405,980	125,213,945	67,387,147	79,908,241	**545,915,313**	7,356	290,926	363,891,933	2,171,191	**366,361,406**	**179,553,907**
**Incremental value**											
**Cervical Screening**	263	304	62	73	**702**	**-28**	**0**	**3,741**	**-378,256**	**-374,542**	**375,244**
**Cervical screening and HPV vaccination**	705	917	166	197	**1,985**	**-124**	**0**	**11,807**	**-2,041,484**	**-2,029,801**	**2,031,786**
**HPV Vaccination**	586	779	138	163	**1,667**	**-112**	**0**	**10,112**	**-1,869,117**	**-1,859,116**	**1,860,783**

**Fig 1:**
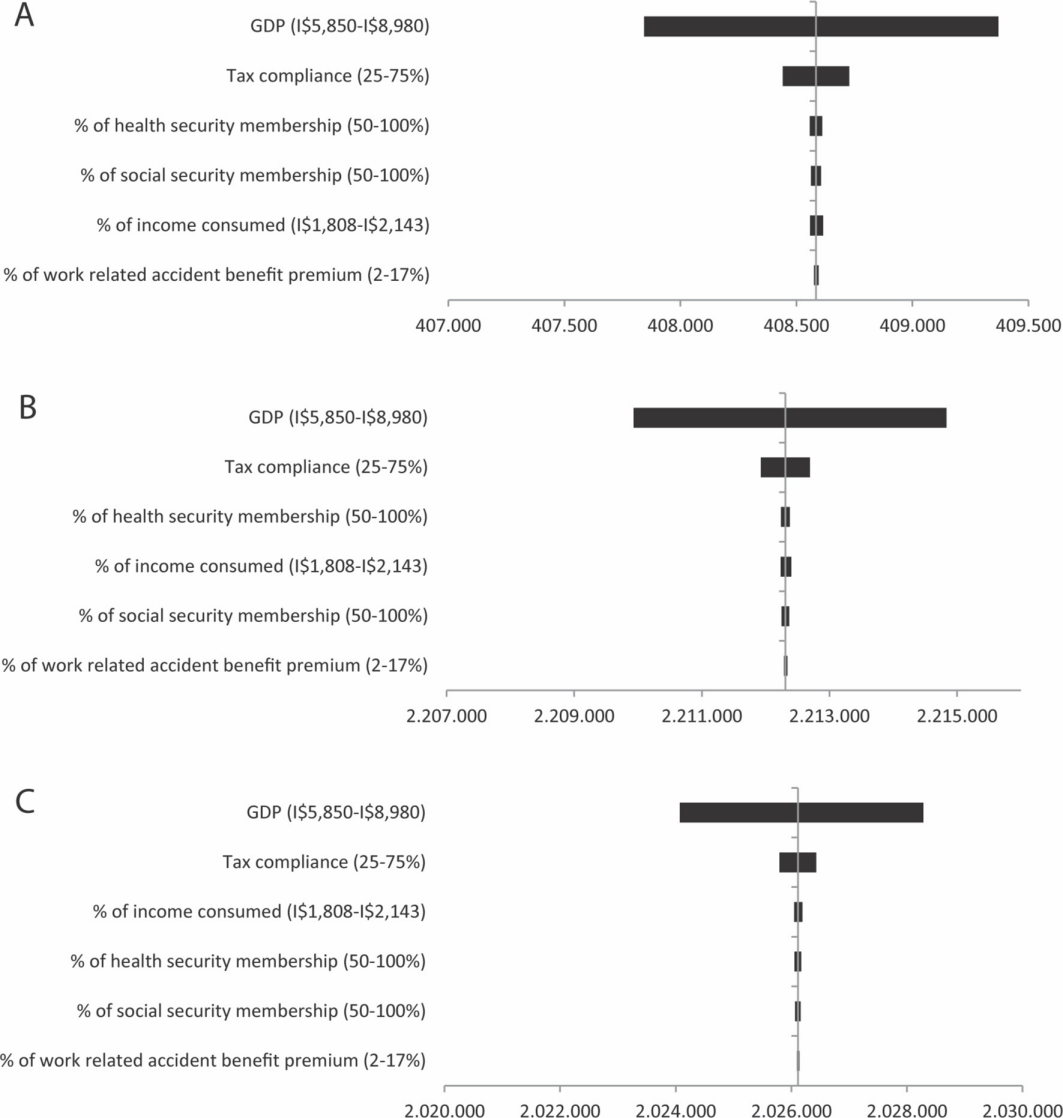
Univariate sensitivity analysis for cervical cancer prevention strategies including cervical screening alone (A); cervical screening and HPV vaccination (B); and HPV vaccination alone (C).

Fig 1 illustrates the results of the univariate sensitivity analyses evaluating the influence of several input parameters on the NPV. The analysis shows that inflation and discount rate are the most influential parameters in this study [data not shown]. Further analysis illustrates that NPV is also sensitive to GDP (as measure of earnings) and tax compliance. Health and social security membership, percentage of earnings consumed, and percentage of work-related accident premium are considered as non-influential parameters to the NPV.

## Discussion

Implementation of population-based healthcare intervention unambiguously results in fiscal costs due to changes in morbidity and mortality that influence national accounts over the lifetime of the cohort. However, similar to other public investments, investment in healthcare interventions may also result in measurable, long-term fiscal benefits (i.e. tax revenue) attributed to changes in population health that may outweigh or considerably offset the costs [52]. In this study we evaluated how different levels of investment in HPV prevention influence the Indonesian governments’ fiscal accounts. The study showed that investing in HPV vaccination in combination with cervical screening yields long-term fiscal benefits for the Indonesian government. These results are supplementary to a previously published study on the cost-effectiveness of cervical cancer prevention strategies in Indonesia [24]. Both studies suggest that cervical cancer prevention strategies are not only beneficial for the health of Indonesian women, they also improve the efficiency of healthcare resource use and have a long-term fiscal benefit for the government by increasing the quality and quantity of the human capital and thus by increasing the tax base in the country compared to not investing in HPV prevention.

The main clinical benefit of cervical cancer prevention programmes is the reduction of morbidity and mortality [16,53–55]. In traditional health-economic analyses the comparative clinical benefits and associated costs would be reflected in the incremental cost effectiveness ratio (ICER) in terms of cost per quality-adjusted survival as a result of vaccination. The latter ratio responds to the question of which is the most efficient use of healthcare resources for a given healthcare budget. A cost-effectiveness analysis may also quantify productivity losses from premature mortality and/or absenteeism or presentism and thus reflect the benefits from a societal perspective which are distinct from benefits accrued to government. In the model described here we apply the tax burden to reflect the proportion of lifetime earnings that are transferred to government to reflect fiscal benefits.

The underlying assumption of taking a broader economic analytic perspective with emphasis on fiscal effects is that changes in health status or the prevention of infectious diseases have several external effects. The benefits of HPV prevention imply that more women who would be able to work thus, gain more earnings and pay more taxes and security premiums for the government. As a result of this, it is expected that the prevention of HPV infection and HPV-related diseases will yield increased governmental fiscal revenues from (direct and indirect) taxes and also (health and social) security insurance premium. Moreover, there are significant cost-savings resulting from reduced need for cervical cancer treatment which is captured in conventional cost-effectiveness analyses and also demonstrated by application of a “government-perspective” framework to assess different HPV investment strategies.

A key limitation of this type of study is that it is largely dependent on the mathematical modeling of long-term clinical, economic and fiscal parameters. Although the population–based model included high-quality clinical-trial data, including vaccine and VIA screening efficacy on incidence and mortality, further model validation using observed-country-specific data could still further assure the outcomes of the model. However, the Indonesia-specific observational information is not available. Such further validation and comparing our model with more sophisticated models from other countries with better data availability remains an option for future research.

Some of the key parameters dynamically change over time in the real life. Sensitivity analyses may address some of the uncertainty around these parameters. However, further observational data may be needed to assess the real-life fiscal effect of HPV prevention programmes. In addition, the current analysis does not take into account the effects of herd immunity, inequalities in access to health-care services due to socio-economic and regional differences, further distinctions in specific stages of both pre-cancer and cervical cancer, nor the benefits that HPV vaccination may have in terms of (i) protection of males and (ii) prevention of genital warts.

By conducting a broader economic analysis, evidence is produced to inform analyses regarding the cross-sectorial allocation of resources and perhaps the transfer of funds from other sectors of the public economy to universal population-based vaccination and screening programmes. The evidence generated in this study may address the affordability of implementing an HPV prevention program in Indonesia. Our study results suggest that investments in HPV prevention programmes may generate epidemiological benefits that translate into health-economic and fiscal benefits for the Indonesian government that may fully offset the investment costs and thus may have a positive impact on national accounts in the long-run.
